# A *rhlI* 5′ UTR-Derived sRNA Regulates RhlR-Dependent Quorum Sensing in Pseudomonas aeruginosa

**DOI:** 10.1128/mBio.02253-19

**Published:** 2019-10-08

**Authors:** Maureen K. Thomason, Maya Voichek, Daniel Dar, Victoria Addis, David Fitzgerald, Susan Gottesman, Rotem Sorek, E. Peter Greenberg

**Affiliations:** a Department of Microbiology, University of Washington, Seattle, Washington, USA; b Department of Molecular Genetics, Weizmann Institute of Science, Rehovot, Israel; c Laboratory of Molecular Biology, CCR, NCI, National Institutes of Health, Bethesda, Maryland, USA; Institut Pasteur

**Keywords:** term-seq, transcriptome, small RNA, Hfq, pyoverdine

## Abstract

*N*-Acyl homoserine lactone (AHL) quorum sensing (QS) controls expression of over 200 genes in Pseudomonas aeruginosa. There are two AHL regulatory systems: the LasR-LasI circuit and the RhlR-RhlI system. We mapped transcription termination sites affected by AHL QS in P. aeruginosa, and in doing so we identified AHL-regulated small RNAs (sRNAs). Of interest, we noted that one particular sRNA was located within the *rhlI* locus. We found that *rhlI*, which encodes the enzyme that produces the AHL *N*-butanoyl-homoserine lactone (C4-HSL), is controlled by a 5′ untranslated region (UTR)-derived sRNA we name RhlS. We also identified an antisense RNA encoded opposite the beginning of the *rhlI* open reading frame, which we name asRhlS. RhlS accumulates as wild-type cells enter stationary phase and is required for the production of normal levels of C4-HSL through activation of *rhlI* translation. RhlS also directly posttranscriptionally regulates at least one other unlinked gene, *fpvA*. The asRhlS appears to be expressed at maximal levels during logarithmic growth, and we suggest RhlS may act antagonistically to the asRhlS to regulate *rhlI* translation. The *rhlI*-encoded sRNAs represent a novel aspect of RNA-mediated tuning of P. aeruginosa QS.

## Abstract (precis)

The opportunistic human pathogen Pseudomonas aeruginosa possesses multiple quorum sensing systems that regulate and coordinate production of virulence factors and adaptation to different environments. Despite extensive research, the regulatory elements that play a role in this complex network are still not fully understood. By using several RNA sequencing techniques, we were able to identify a small regulatory RNA we named RhlS. RhlS increases translation of RhlI, a key enzyme in the quorum sensing pathway, and represses the *fpvA* mRNA encoding one of the siderophore pyoverdine receptors. Our results highlight a new regulatory layer of P. aeruginosa quorum sensing and contribute to the growing understanding of the role regulatory RNAs play in bacterial physiology.

## INTRODUCTION

The opportunistic pathogen Pseudomonas aeruginosa, like many bacteria, has the ability to sense its population density and respond to environmental changes by initiating a gene regulatory system termed quorum sensing (QS). In proteobacteria like P. aeruginosa, QS commonly involves diffusible *N*-acylhomoserine lactone (AHL) signaling molecules that are recognized by corresponding transcription factors. When the population density, and thus signal concentration, reaches a critical threshold, a coordinated, population-wide shift in gene expression occurs. This facilitates P. aeruginosa adaptation to its environment (1, 2). In P. aeruginosa, there are two AHL QS systems: the Las system and the Rhl system. The Las system consists of LasI, which catalyzes synthesis of *N*-3-oxo-dodecanoyl-homoserine lactone (3OC12-HSL), and the 3OC12-HSL-dependent transcription factor LasR. The Rhl system consists of RhlI, the *N*-butanoyl-homoserine lactone (C4-HSL) synthase, and the C4-HSL-dependent transcription factor RhlR. Together these two QS circuits activate over 200 genes (3–7). Although much is known about direct control of P. aeruginosa genes by LasR and RhlR, less is known about QS-mediated posttranscriptional regulation by noncoding RNA elements.

Over the last two decades, we have learned that noncoding RNA is critical for the posttranscriptional control of gene expression. Posttranscriptional regulation by small regulatory RNAs (sRNAs) is known to occur by two mechanisms: by direct base pairing to target mRNAs or by binding to proteins. In the class of base-pairing sRNAs, *trans*-encoded sRNAs base pair with limited complementarity to target mRNAs mediated by the RNA chaperone protein Hfq and recently discovered ProQ domain-containing proteins (reviewed in reference 8). Antisense RNAs (asRNAs), the second type of base-pairing sRNA, are encoded on the strand opposite to that encoding the mRNA and base pair with the mRNA, generally in the absence of protein chaperones, to regulate either translation or stability of their target mRNA (reviewed in references 9 and 10).

RNA-mediated control of transcription or translation can also involve riboswitches or thermosensors found within the 5′ untranslated regions (UTRs) of some genes or operons. They control gene expression or translation by directly binding metabolites or signaling molecules, by sensing pH, or by sensing changes in temperature (11, 12). In the absence of a canonical riboswitch, the secondary structure of the RNA in the 5′ UTR can on occasion regulate translation by sequestering access to a ribosome-binding site (RBS). For example, in P. aeruginosa the sRNA PhrS regulates expression of *pqsR*, which codes for a transcriptional activator, by binding the RBS of an upstream open reading frame (uORF). The binding alters the RNA structure to activate translation of the uORF, which by translational coupling leads to *pqsR* translation (13). Recent studies suggest riboswitches, 5′ UTRs, sRNAs, and asRNAs can regulate each other, thus forming complex regulatory networks (14, 15).

Given the complexity of RNA-based regulation, it is not surprising that sRNAs and QS are intricately linked. For example, similar to other bacteria, P. aeruginosa Hfq mutants grow abnormally and are attenuated for virulence (16). In the context of QS, transcript profiling showed that Hfq influences expression of 72 QS-activated genes (16–18). More recently, a high-resolution transcriptome sequencing (RNA-seq) study identified a number of sRNAs, including two that were induced by the LasR QS system (19), while another study identified the sRNA PhrD as a positive regulator of *rhlR* (20). Independent of sRNA-based regulation, two RNA thermometers were shown to control expression of the RhlR-activated *rhlAB* operon, as well as *lasI* (21). Although P. aeruginosa genome-wide RNA-seq studies have identified hundreds of potential sRNAs and asRNAs (18, 19, 22–25, 28), none have focused specifically on identifying regulatory RNA elements controlled by QS.

We have used term-seq (26) to quantitatively map 3′ ends of RNA in P. aeruginosa and identify those ends affected by QS. We did not identify any QS-responsive riboswitches, but we did identify a number of sRNAs not previously associated with QS. There was a strongly QS-induced transcription termination site in the 5′ UTR of *rhlI.* Follow-up investigations led us to describe an sRNA we name RhlS, which is derived from the 5′ UTR of *rhlI*. RhlS activates translation of *rhlI* and induces C4-HSL production, which can be partially complemented in *trans*. RhlS also acts posttranscriptionally in *trans* to regulate the *fpvA* mRNA, which encodes a siderophore receptor (27). Furthermore, the term-seq analysis revealed an antisense RNA opposite *rhlI*. We call this antisense RNA asRhlS and present evidence that RhlS may act as an asRhlS antagonist in P. aeruginosa.

## RESULTS

### Identification of QS-induced RNA 3′ ends by term-seq analysis.

**FIG 1:**
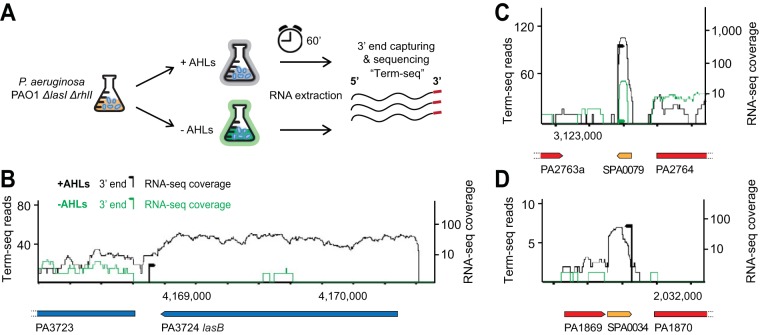
Term-seq method to identify QS-regulated RNAs. (A) Schematic representation of the experimental setup. (B) Expression and termination of the *lasB* elastase-encoding gene is induced in the presence of AHLs in the PAO1 *ΔlasI ΔrhlI* mutant (MPK0493). (C) Expression of the SPA0079 sRNA (28) increases upon addition of AHLs. (D) Expression of the SPA0034 sRNA (28) increases upon addition of AHLs. For panels B to D, the number of reads at the term-seq site represents the average of normalized strand-specific term-seq reads in the dominant term-seq position (see Text S1). Black arrowheads indicate 3′ ends with AHLs (+AHLs) measured by term-seq (the directionality of the arrowhead is opposed to the expressed strand), while green ones indicate 3′ ends without AHLs (−AHLs). Black lines indicate +AHL RNA-seq reads, and green lines indicate −AHLs RNA-seq reads. Arrows at the bottom of each figure indicate gene annotations, and dashed lines indicate gene annotations that extend beyond that depicted.

We mapped the RNA 3′ termini, representing to a large extent the transcription termination sites (TTSs) in a P. aeruginosa PAO1 LasI, RhlI (AHL synthesis) mutant incubated with or without added 3OC12-HSL and C4-HSL by using term-seq as diagrammed in Fig. 1A and described in detail in the supplemental material (see Text S1). We identified a total of 804 TTSs associated with annotated P. aeruginosa genes or operons (see Table S1, tab A, in the supplemental material).

10.1128/mBio.02253-19.1 TEXT S1 Supplemental materials and methods. Download Text S1, PDF file, 0.3 MB. Copyright © 2019 Thomason et al. 2019 Thomason et al. This content is distributed under the terms of the Creative Commons Attribution 4.0 International license.

10.1128/mBio.02253-19.9 TABLE S1 (Tab A) Transcription termination sites (TTS) associated with P. aeruginosa PAO1 genes. (Tab B) Differential expression analysis for reproducible term-seq sites with and without AHLs. Base mean, fold change, and adjusted *P* value were calculated using DESeq2. (Tab C) Predicted targets of RhlS according to TargetRNA2 analysis. (Tab D) Strains, plasmids, and oligonucleotides used in this study. Download Table S1, XLSX file, 0.6 MB. Copyright © 2019 Thomason et al. 2019 Thomason et al. This content is distributed under the terms of the Creative Commons Attribution 4.0 International license.

**FIG 2:**
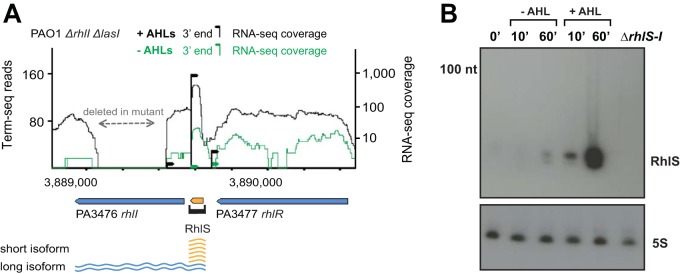
Analysis of a QS-regulated RNA element in the 5′ UTR of *rhlI.* (A) RNA-seq and term-seq data for the *rhlI* locus in PAO1 *ΔlasI ΔrhlI* (MPK0493). Expression and termination of RhlS (orange arrow) located in the 5′ UTR of *rhlI* increases substantially upon addition of AHLs, producing two alternative isoforms from the same locus. The gap in RNA-seq coverage within *rhlI* is due to the deletion of the ORF. The number of reads at the term-seq site (black pillar) was determined as in Fig. 1 (Text S1). The RNA-seq coverage shown is not strand-specific. (B) Northern analysis of RhlS expression in PAO1 *ΔlasI ΔrhlI* (MPK0493). Overnight cultures of PAO1 Δ*lasI* Δ*rhlI* were grown as described (Text S1). At an optical density (OD_600_) of ∼0.8, the cultures were split and no AHL or both C4-HSL (10 μM) and 3OC12-HSL (2 μM) were added to the cultures. Cells were harvested after 10 and 60 min, RNA was extracted and 10 μg total RNA was analyzed by Northern blotting with a ^32^P-labeled oligonucleotide specific to RhlS or 5S as a loading control. RNA from the *ΔrhlS-I* (MPK0627) strain collected at OD_600_ of 2.0 was used as a control for band specificity.

In addition, we identified 21 RNA termini whose expression levels were elevated or reduced by AHLs (Table 1; Table S1, tab B). We believe this is an underrepresentation of QS-regulated genes because of our stringent analysis criteria and limited experimental conditions. Most of the sites affected by AHLs correspond to the 3′ ends of known QS-regulated genes. As an example, term-seq reads depicting the 3′ end of *lasB*, which codes for the QS-induced elastase enzyme, were much more abundant in cells incubated with AHLs than in cells without AHLs (Fig. 1B; Table S1, tab B). We also identified AHL-controlled TTSs for a number of previously identified sRNAs not known to be associated with QS (Table 1; Table S1, tab B). Among those regulated by AHLs, two previously identified sRNAs (28) are shown in Fig. 1C and D. Interestingly, one of these sRNAs, designated SPA0034 (28), is located downstream of a known QS-activated gene, PA1869 (5) (Fig. 1D), and might be a 3′ UTR-derived sRNA similar to those identified in other bacterial species (29–32). Notably, we identified a premature transcription termination signal in the 5′ UTR of *rhlI* (Fig. 2). We chose to focus on the AHL-induced *rhlI* 5′ UTR for two reasons. The expression of this 5′ UTR showed a substantial dependence on AHLs (Table 1), and *rhlI* codes for the C4-HSL QS signal synthase, which is itself QS activated and required for a full QS response.

**TABLE 1:** Known small RNAs identified by term-seq as differentially regulated by AHLs

sRNA name	Flanking genes (5′/3′)	3′ end position^a^	sRNA strand	Fold change^b^	Comments and reference(s)
RhlS/SPA104	*rhlR*/*rhlI*	3889777	−	55.68	Fig. 2A (28)
SPA0116	PA2768/PA2769	3127925	+	28.56	PA2769, known to be QS regulated (28, 54)
SPA0079	PA2763/PA2764	3123367	−	18.05	Fig. 1C (28)
SPA0034	PA1869/PA1870	2031856	+	3.06	Fig. 1D (28)
SPA0080	PA2789/PA2790	3147657	+	2.94	28
AmiL	*amiE*/PA3367	3778033	−	2.92	18
pant90	PA0806/PA0807	884182	−	2.26	23

a Indicates position of the 3′ end signal in the PAO1 genome.

b Indicates fold change with or without AHLs determined as differentially expressed if they changed by more than 2-fold with a *P* value of <0.05.

### Transcription termination in the 5′ UTR of *rhlI*.

**FIG 3:**
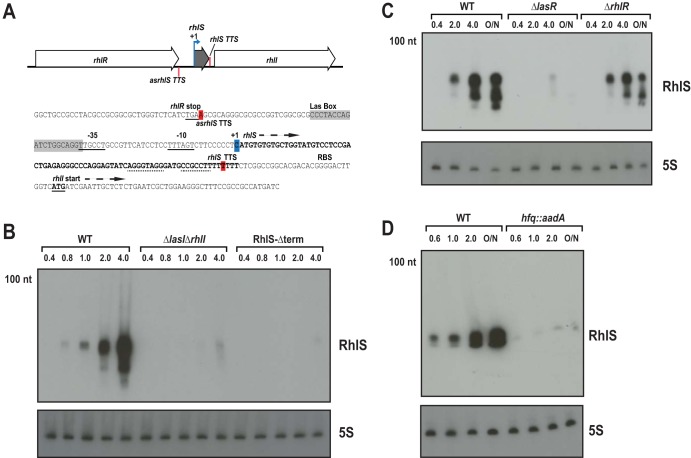
Expression of RhlS as a function of culture growth, LasR, and Hfq. (A) Schematic and sequence of *rhlR-rhlS-rhlI* locus. Blue arrow indicates the +1 site of transcription, and red bars indicate the termination points. The *rhlS-rhlI* −10 and −35 sequences are underlined and the Las box is boxed in gray. Dashed lines indicate the putative RhlS Rho-independent terminator. (B) Northern analysis of RhlS. WT PAO1 (MPK0409), PAO1 *ΔlasI ΔrhlI* (MPK0493), or the RhlS-Δterm mutant (MPK0555) was grown as described (Text S1). Samples were collected and processed for Northern analysis as in Fig. 2. (C) Influence of LasR and RhlR on RhlS levels. WT PAO1 (MPK0409) and the isogenic PAO1 Δ*lasR* (MPK0426) and PAO1 Δ*rhlR* (MPK0428) mutants were grown as in panel B, and samples were processed for Northern analysis as in Fig. 2. (D) RhlS levels require Hfq. WT PAO1 (MPK0530) and the isogenic *hfq*::*aadA* mutant (MPK0529) were grown as in panel B, and samples were processed for Northern analysis as in Fig. 2. In panels B to D, the numbers above the images of autoradiographs indicated the OD_600_ at which cells were harvested for RNA extraction.

The AHL-induced RNA 3′-end in the *rhlI* UTR is 34 nucleotides upstream of the *rhlI* start codon, and expression of this RNA terminus in AHL-induced cells was over 50 times higher than in uninduced cells (Fig. 2A). Additionally, there is a TTS upstream of *rhlI* that maps to the end of the *rhlR* ORF. Thus, the 3′ end detected in the 5′ UTR of *rhlI* is not likely due to transcriptional read-through from *rhlR*. We analyzed whole transcriptome RNA-seq data and found that AHLs induced expression upstream of the *rhlI* UTR termination signal by about 100-fold over uninduced cells (Fig. 2A). An overrepresentation of RNA-seq reads in the *rhlI* 5′ UTR can also be found in previously published RNA-seq data sets of wild-type P. aeruginosa strain PA14 (19, 33). We also identified an RNA 3′ end in the antisense orientation to the *rhlI* ORF overlapping the sequence of *rhlI*, although expression of this RNA appeared to be very low (Fig. 3A and see Fig. 6B below).

### The 5′ UTR does not appear to encode a C4-HSL riboswitch.

Because term-seq has been used to discover 5′ UTR-derived ribo-regulators that mediate premature transcription termination in other bacteria (26), we asked whether the *rhlI* 5′ UTR might code for a C4-HSL-responsive riboswitch. We first approached this question by using bioinformatics. Neither the PASIFIC (34) nor RFAM (35) predictive structure analysis programs revealed any putative riboswitch-like motifs in the *rhlI* 5′ UTR. While informative, these searches are not exhaustive; therefore, we also addressed this question experimentally. We constructed an E. coli reporter containing the entire *rhlI* 5′ UTR through the first 30 codons fused to *lacZ*. Expression of this construct was arabinose inducible. We found that when expression of the construct was activated with arabinose, there was no effect on β-galactosidase levels when C4-HSL was added (see Fig. S1 in the supplemental material). The results from both the bioinformatics and experimental approaches were inconsistent with the idea that the *rhlI* 5′ UTR is a C4-HSL-responsive riboswitch. We cannot rule out the possibility that this UTR may be responsive to other signaling molecules. However, these results led us to test other possible consequences of early *rhlI* transcription termination.

10.1128/mBio.02253-19.2 FIG S1 The 5′ UTR of *rhlI* does not respond to exogenous C4-HSL as a riboswitch. Overnight cultures of E. coli P_BAD_-*rhlS-rhlI*::*lacZ* (MPK0601) were diluted into 100 ml fresh LB plus 50 mM MOPS in 500-ml baffled flasks and grown at 37°C with shaking. At an OD_600_ of ∼0.4, the cultures were split and either only 10 μM C4-HSL, only 0.2% arabinose, both, or neither were added to each flask. At the indicated times, samples were collected and β-galactosidase levels were determined. Download FIG S1, PDF file, 0.2 MB. Copyright © 2019 Thomason et al. 2019 Thomason et al. This content is distributed under the terms of the Creative Commons Attribution 4.0 International license.

### The 5′ UTR of *rhlI* encodes an sRNA.

We hypothesized the premature termination of the *rhlI* 5′ UTR with added AHLs could generate a stable sRNA and investigated this using Northern blot analysis. As shown in Fig. 2B, we detected a QS signal-induced sRNA that is less than 100 nucleotides in length as early as 10 min after exposure to AHLs. This sRNA accumulated for at least 60 min after AHLs were added to the cells. We did not detect the sRNA when we analyzed a 5′ UTR*-rhlI* deletion mutant, consistent with the conclusion that the sRNA is specific to the *rhlI* locus (Fig. 2B). We have named this sRNA RhlS (RhlI-associated sRNA). The location and size of RhlS are consistent with a previously identified 70-nucleotide P. aeruginosa sRNA (SPA104) that was not known to be AHL induced (28).

We next mapped the 5′ end of RhlS by primer extension (see Fig. S2A in the supplemental material) and the 5′ end of both RhlS and *rhlI* with 5′ RACE (rapid amplification of cDNA ends) (Fig. 3A; Fig. S2B). We found the predominant transcript start site of both RhlS and *rhlI* corresponded to the +1 position of *rhlI* transcription previously reported (36). We also identified a stem-loop structure followed by a polyuridine tract, consistent with a Rho-independent transcriptional terminator sequence immediately upstream of the RNA 3′ end detected in the *rhlI* 5′ UTR sequence (Fig. 3A). By using the boundaries defined by the transcript start site and term-seq, we infer that RhlS is about 70 nucleotides in length. A transcript of this size is consistent with the RhlS band seen by Northern analysis (Fig. 2B). Our data suggest that, in the presence of AHLs, the *rhlI* locus can be transcribed from a single *rhlI* promoter into two isoforms: the long isoform encoding full-length *rhlI* mRNA and, as our RNA-seq data suggest, a more abundant short RhlS resulting from premature transcription termination within the 5′ UTR (Fig. 2A).

10.1128/mBio.02253-19.3 FIG S2 Analysis of transcription start sites identifies a single transcription start for RhlS and *rhlI*. (A) Primer extension analysis. To detect the 5′ end of RhlS, primer VA0001 (corresponding to nucleotides −61 to −42 relative to the *rhlI* start of translation) was end labeled with [γ-^32^P]ATP and incubated with the primer extension enzyme mixture. The *rhlI*-*rhlR* fragment was amplified from PAO1 to generate the sequencing ladder. To resolve the 5′ end of RhlS, primer extension reactions and the DNA sequencing ladder were run on an 8% polyacrylamide–6 M urea gel. * corresponds to the +1 site of RhlS indicated in Fig. 3A. (B) 5′ RACE data for RhlS and *rhlI*. Total RNA isolated from WT PAO1 was subjected to 5’ RACE analysis using the indicated primers. Each row represents an independent clone (and RNA transcript) that was sequenced. Download FIG S2, PDF file, 0.2 MB. Copyright © 2019 Thomason et al. 2019 Thomason et al. This content is distributed under the terms of the Creative Commons Attribution 4.0 International license.

### RhlS is regulated by QS and is dependent on Hfq.

We next examined RhlS production in wild-type P. aeruginosa. Northern blotting showed that RhlS levels in early-logarithmic-phase cells were relatively low, increased in late logarithmic phase, and were at maximal levels in stationary-phase cells (Fig. 3B). There is a LasR binding site in the promoter region of *rhlI*, and *rhlI* is activated strongly by LasR and weakly by RhlR (36). Because the transcript starts for RhlS and *rhlI* appeared to be the same, we hypothesized that RhlS transcription would be activated primarily by LasR and to a lesser extent by RhlR. To test this hypothesis, we monitored RhlS levels in strains deleted for *lasR* and *rhlR* by Northern blotting. As predicted, RhlS levels in the LasR mutant were very low and RhlS was modestly decreased in the RhlR mutant (Fig. 3C). Thus, the increase in RhlS as a function of growth appears to be primarily a consequence of LasR-dependent QS induction.

A previous report showed that there was a marginal effect of Hfq on *rhlI* mRNA levels (17). We asked whether Hfq might affect RhlS and the *rhlI* transcript differently. In fact, Northern blotting showed very low levels of RhlS in an Hfq mutant in comparison to levels in wild-type cells (Fig. 3D). This is consistent with the conclusion that the two transcripts produced from the *rhl* locus have different requirements for Hfq. RhlS levels are drastically altered in the absence of Hfq, while *rhlI* levels are only slightly altered (∼1.5-fold decreased in an *hfq* mutant) (17).

### Disruption of the RhlS terminator reduces C4-HSL production.

**FIG 4:**
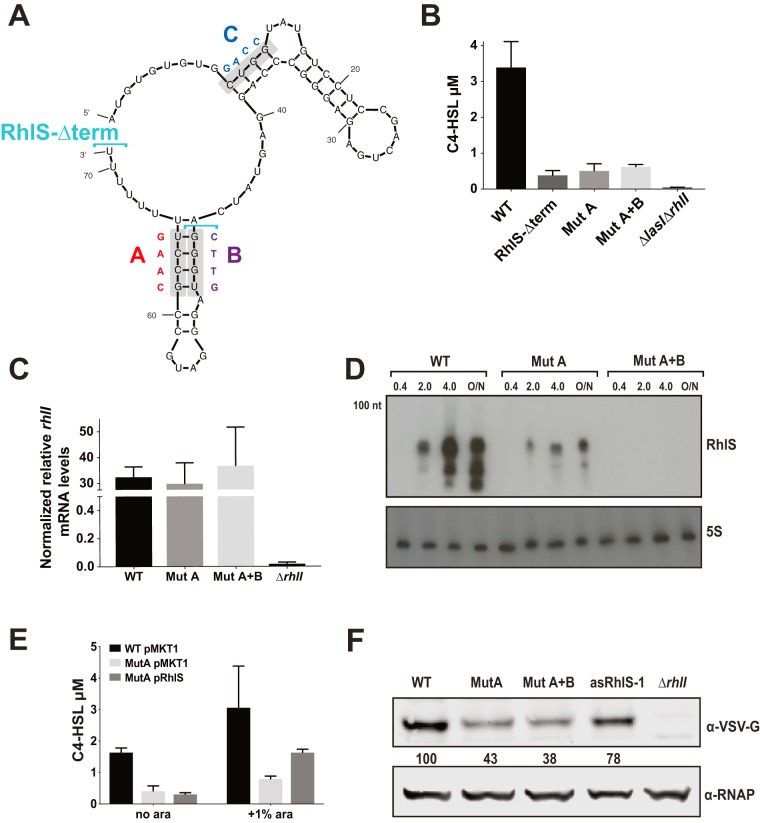
RhlS regulation of RhlI translation and C4-HSL production. (A) Predicted Mfold (37) structure of the RhlS sRNA. Gray boxes indicate positions and red letters indicate nucleotides changed for MutA, MutA+B and MutC point mutants. Cyan brackets indicate nucleotides deleted in the RhlS-Δterm mutant. (B) C4-HSL levels in RhlS point mutant strains compared to wild type. Supernatant was collected from overnight cultures of WT PAO1 (MPK0409) and the isogenic RhlS-Δterm mutant (MPK0555), MutA mutant (MPK0576), MutA+B mutant (MPK0619), and as a control, the PAO1 *ΔlasI ΔrhlI* mutant (MPK0493) after 24 h. C4-HSL was extracted and measured by using the C4-HSL bioassay. Values are the means of three biological and two technical replicates, and error bars are standard deviations. (C) Levels of *rhlI* mRNA in the wild-type PAO1, MutA, and MutA+B strains was determined by quantitative reverse transcription-PCR (qRT-PCR) using primers specific to the *rhlI* open reading frame. The amount of *rhlI* mRNA was calculated with a standard curve and normalized to levels of the housekeeping control gene *groEL*. Values are the mean of two biological and two technical replicates. Bars are standard deviations. (D) Levels of RhlS in the point mutant strains. The wild type and the isogenic MutA and MutA+B mutants were grown and Northern analysis performed as in Fig. 3. (E) RhlS can partially complement the MutA C4-HSL production defect when provided in *trans*. Wild-type PAO1 and the PAO1 MutA point mutant were transformed with the empty pMKT1 vector or pRhlS. Single colonies were inoculated into LB plus 50 mM MOPS and grown in the presence or absence of 1% l-arabinose at 37°C. After 24 h, C4-HSL was extracted and measured. Results are means of three biological and two technical replicates, and bars are the standard deviation. (F) Western blot analysis of VSV-G epitope-tagged RhlI. Strains: WT-RhlI-VSV-G (MPK0698), MutA-VSV-G (MPK0689), MutA+B-VSV-G (MPK0697), asRhlS-1-VSV-G (MPK0687), and *ΔrhlS-rhlI* (MPK0627). Cells were grown as in panel B. RhlI levels were normalized to the corresponding RNA polymerase band and are presented as a percentage of wild type. The image is representative of three independent experiments.

From the term-seq analysis, we estimate that RhlS is the predominant transcript derived from the *rhII* promoter, with full-length *rhlI* mRNA accounting for between 3 and 15% of the total at steady state. RNA structure prediction using Mfold (37) showed a predicted structure with a 5′ end hairpin and a Rho-independent terminator (Fig. 4A). We hypothesized that disruption of the Rho-independent terminator should result in increased transcriptional readthrough producing more full-length *rhlI* transcript and thus elevated levels of C4-HSL. We tested this hypothesis by constructing a strain where the Rho-independent terminator is deleted (RhlS-Δterm). Surprisingly, the amount of *rhlI* mRNA in this mutant was similar to that in wild-type cells (see Fig. S3 in the supplemental material), but RhlS levels were lower (Fig. 3B). The deletion may have eliminated an essential Hfq binding site present in the terminator (38). The terminator deletion mutant also produced almost 10-fold less C4-HSL than the wild-type (Fig. 4B). Because the wild type and mutant had roughly equivalent levels of *rhlI* mRNA, we surmise that the defect in C4-HSL production in the terminator mutant is due to RhlS-mediated posttranscriptional regulation of *rhlI*.

10.1128/mBio.02253-19.4 FIG S3 Levels of the *rhlI* mRNA are similar in the WT and the RhlS-Δterm mutant. Single colonies of WT PAO1 and the RhlS-Δterm mutant (MPK0555) were grown for 24 h in 10 ml LB plus 50 mM MOPS in 50-ml flasks at 37°C with shaking. Total RNA was extracted, cDNA was generated, and mRNA levels were measured as in Fig. 4C. Results are means of two independent experiments with two technical replicates each, and error bars show standard deviations. Download FIG S3, PDF file, 0.3 MB. Copyright © 2019 Thomason et al. 2019 Thomason et al. This content is distributed under the terms of the Creative Commons Attribution 4.0 International license.

To gain insight into whether the terminator structure or sequence of RhlS was responsible for the phenotype exhibited by the terminator deletion mutant, we made point mutations in the RhlS sequence. These point mutations (Fig. 4A, Mut A) should disrupt the terminator structure while leaving the sequence largely intact. We also made a compensatory mutation that should restore base pairing in the terminator and thus recover the structure (Fig. 4A, Mut A+B). The structure-disrupting mutant and the compensating mutant had similar levels of *rhlI* mRNA but produced low levels of C4-HSL and low levels of RhlS (Fig. 4B to D). These data are consistent with the conclusion that the sequence of the RNA in the RhlS terminator region is essential for RhlS function, and RhlS is important for C4-HSL production but not *rhlI* mRNA levels. It is conceivable that Hfq binds to RhlS in this region to promote the sRNA-mediated posttranscriptional regulation of *rhlI*.

The defect in C4-HSL production can be partially restored by *trans*-complementation of RhlS on a multicopy plasmid (pRhlS) in the RhlS terminator structure mutation strain (Fig. 4E). Arabinose-induced P. aeruginosa (pRhlS) had RhlS transcript levels similar to those of wild-type cells indicating expression from the pRhlS plasmid is physiologically relevant (see Fig. S4A in the supplemental material). Additionally, when an arabinose-induced promoter-*rhlI* fusion was integrated in the Escherichia coli chromosome, the cells produced micromolar amounts of C4-HSL similar to those of wild-type P. aeruginosa (Fig. S4B). This indicates the mechanism by which RhlS induces *rhlI* translation is either conserved between E. coli and P. aeruginosa or contained entirely within the RhlS-*rhlI* locus. Our data suggest that high levels of RhlS are required to maintain wild-type levels of C4-HSL but are not required for *rhlI* mRNA accumulation. In our experiments, *rhlI* mRNA levels remain unchanged regardless of RhlS expression, while C4-HSL levels vary in mutants expressing less RhlS.

10.1128/mBio.02253-19.5 FIG S4 Ectopic expression of RhlS is physiologically relevant. (A) RhlS levels in the *rhlS-rhlI* mutant (MPK0627) containing pRhlS or the vector pMKT1 are similar to WT PAO1. For the mutants, overnight cultures were diluted to an OD_600_ of ∼0.005 in LB plus 50 mM MOPS, and at an OD_600_ of ∼0.4, the cultures were split and either no or 1% l-arabinose was added. Samples were collected at the indicated times after addition of arabinose. For wild-type PAO1 cells were grown similarly but arabinose was not added and culture densities at sampling times are shown. For all samples, RNA was extracted and analyzed by Northern analysis as in Fig. 2B. (B) Levels of C4-HSL produced by E. coli with a chromosomal insertion of *rhlI* compared to levels produced by wild-type P. aeruginosa PAO1. Single colonies of PAO1 or E. coli PM1205-P_BAD_-*rhlS-rhlI* (MPK0603) were used to inoculate 10 ml LB plus 50 mM MOPS in 50-ml flasks and grown at 37°C in the presence of 1% l-arabinose for 18 h, C4-HSL was extracted and measured as in Fig. 4B. Data are the means of three biological replicates, and error bars are standard deviations. Download FIG S4, PDF file, 0.1 MB. Copyright © 2019 Thomason et al. 2019 Thomason et al. This content is distributed under the terms of the Creative Commons Attribution 4.0 International license.

### RhlS controls translation of *rhlI* mRNA.

Because mutants that produce normal levels of *rhlI* mRNA but low levels of RhlS produce low levels of C4-HSL, we hypothesized that RhlS might stimulate translation of *rhlI* mRNA. To test this hypothesis, we incorporated a C-terminal vesicular stomatitis virus glycoprotein G (VSV-G) epitope tag at the native *rhlI* locus such that it encoded an RhlI-VSV-G polypeptide. We examined levels of this tagged RhlI in our RhlS mutant strains by Western blotting (Fig. 4F). Consistent with the hypothesis, RhlI levels were lower in the RhlS mutants than in the wild type. We confirmed that RhlI-VSV-G was active by showing the RhlI-VSV-G-tagged version produced C4-HSL levels comparable to those of the native RhlI protein (see Fig. S5 in the supplemental material). Thus, RhlS appears to affect translation but not the *rhlI* mRNA.

10.1128/mBio.02253-19.6 FIG S5 The RhlI-VSV-G epitope tag does not alter C4-HSL production. Single colonies of WT RhlI-VSV-G (MPK0698), RhlS Mut A-VSV-G (MPK0689), RhlS MutA+B VSV-G (MPK0697) and the asRhlS-1-VSV-G (MPK0687) strains were grown for 24 h in 10 ml LB plus 50 mM MOPS in 50-ml flasks at 37°C with shaking. C4-HSL was measured as described in Fig. 4B. Data are from three biological and two technical replicates for each strain, and error bars are standard deviations. Download FIG S5, PDF file, 0.1 MB. Copyright © 2019 Thomason et al. 2019 Thomason et al. This content is distributed under the terms of the Creative Commons Attribution 4.0 International license.

### Direct regulation of *fpvA* by RhlS.

**FIG 5:**
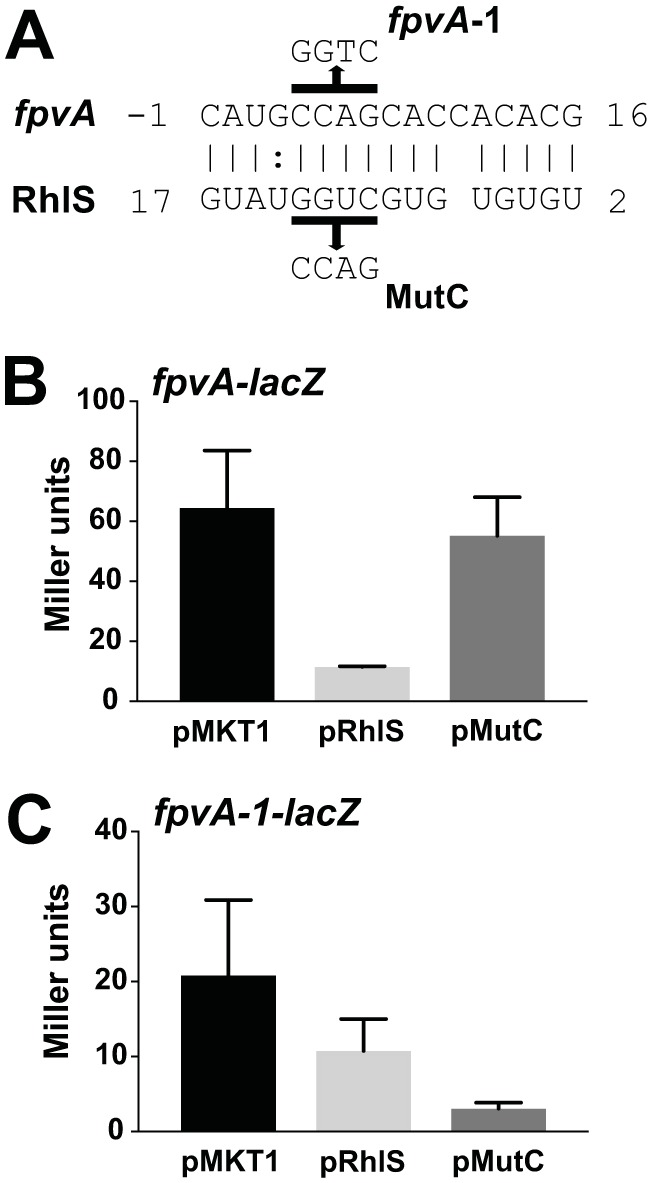
RhlS regulation of FpvA production. (A) TargetRNA2 (39) RhlS and *fpvA* base-pairing predictions. The lines over and under the sequences show mutations we constructed and are relative to the start codon for *fpvA* and the +1 site of RhlS transcription. (B) Negative regulation of *fpvA-lacZ* by RhlS. The reporter strain PM1205 *fpvA-lacZ* (MPK0704) was transformed with the control vector pMKT1, pRhlS, or the pMutC mutant derivative. β-Galactosidase levels were assayed after 3 h of induction with 0.4% arabinose. The averages of three independent assays are shown, and error bars are standard deviations. (C) Disruption and restoration of base pairing between RhlS and *fpvA*. The pMKT1 vector, pRhlS or pMutC plasmids were transformed into the PM1205 *fpvA-1-lacZ* mutant strain (MPK0712), which carries compensatory mutations to restore regulation to MutC. β-Galactosidase levels were assayed as in panel B.

It seemed possible that RhlS might affect translation of genes other than *rhlI*. To assess this possibility, we used the base-pairing prediction algorithm TargetRNA2 (39) to search for genomic regions that might base pair with RhlS (Table S1, tab C). The hit with the most extensive base-pairing complementarity was a 16 nucleotide region in the first five codons of the *fpvA* open reading frame that pairs with RhlS (Fig. 5A). The *fpvA* gene product is a receptor for the P. aeruginosa siderophore pyoverdine (27).

To test the hypothesis that RhlS controls *fpvA* expression, we created an arabinose-inducible translational reporter containing the *fpvA* 5′ UTR through the first 25 codons of the *fpvA* ORF fused in frame to *lacZ* and placed this construct on the chromosome of E. coli. In this construct, we either expressed RhlS on an arabinose-inducible plasmid (pRhlS) or included the empty vector (pMKT1). Levels of β-galactosidase in arabinose-grown cells containing pRhlS were about 25% of the levels in cells without RhlS (Fig. 5B). To test whether the RhlS repression of *fpvA* was by direct base pairing via the 16-nucleotide region identified in the TargetRNA2 analysis, we did the following: We first changed the RhlS sequence to disrupt base pairing with *fpvA*. When we used a plasmid (pMutC) expressing the mutant RhlS in place of wild-type RhlS, *fpvA-lacZ* expression was not repressed (Fig. 5B). We then constructed an arabinose-inducible *fpvA-1-lacZ* reporter with a mutation that compensated for the pMutC mutation, and this restored *lacZ* repression (Fig. 5C). We note that levels of *fpvA-1-lacZ* expression in cells containing the compensatory mutation are lower than those in cells containing the wild-type *fpvA-lacZ* fusion. We believe this may be due to the fact that changing the sequence of the first few *fpvA* codons decreased translation efficiency. These experiments indicate that RhlS can serve to regulate *fpvA* mRNA translation by a direct base-pairing mechanism, and they point to a link between QS and iron homeostasis in P. aeruginosa.

### The *rhlI* antisense RNA may be involved in regulating *rhlI* translation.

**FIG 6:**
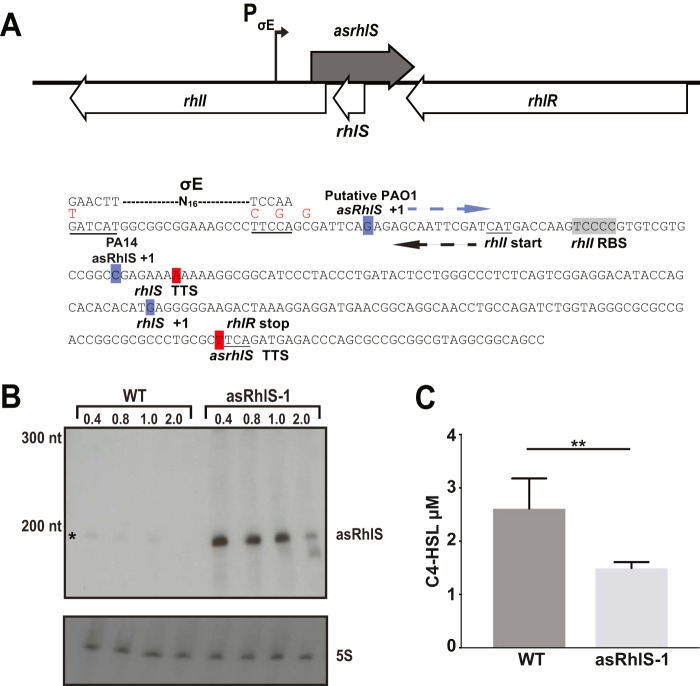
An antisense RNA is encoded in the *rhlI* locus and regulates C4-HSL production. (A) Schematic and sequence of the asRhlS promoter region, including the overlap with RhlS and *rhlI*. Blue boxes indicate the putative +1 of transcription for PAO1 and the known +1 for PA14 and red box indicates the asRhlS termination point. The dashed line indicates a predicted σ^E^ site for asRhlS and the consensus sequence is indicated above. Red letters are the nucleotide changes for the asRhlS-1 mutant. For reference and orientation the *rhlI* start codon, *rhlS* start and stop, and *rhlR* stop codon are indicated. The ribosome binding site (RBS) for *rhlI* is shaded gray. (B) The asRhlS-1 mutation increases expression of asRhlS. WT PAO1 and the isogenic asRhlS-1 promoter mutant (MPK0637) were grown and processed for Northern analysis as in Fig. 3 with an oligonucleotide specific to asRhlS. * indicates the wild-type asRhlS transcript band. (C) C4-HSL levels are reduced in the asRhlS-1 promoter strain. Wild-type PAO1 (MPK0409) and asRhlS-1 (MPK0637) were grown and C4-HSL levels were determined as in Fig. 4. Values are means of three biological and two technical replicates for each strain, and error bars are standard deviations. ** indicates *P* < 0.005 using an unpaired *t* test with Welch’s correction.

As mentioned earlier, we detected low levels of an *rhlI* antisense RNA in our term-seq analysis (Fig. 3A). A map showing the chromosomal region encoding this asRNA with the term-seq detected TTS is shown in Fig. 6A. We confirmed the existence of the antisense RNA, which we call asRhlS, by Northern blot analysis. Expression of asRhlS was at peak, although still low, abundance during logarithmic growth of P. aeruginosa (Fig. 6B). The size of the asRhlS band is slightly less than 200 nucleotides. Because asRhlS is in low abundance, we were unable to map the exact transcription start site by 5′ RACE. However, based on the TTS determined by our term-seq analysis and the size of the band on the Northern blot, we identified a putative TSS (transcription start site) for asRhlS (Fig. 6A; see Fig. S6 in the supplemental material). We predict asRhlS overlaps the beginning of the *rhlI* ORF as well as the entire *rhlI* 5′ UTR. Previous transcription start site mapping identified an antisense TSS in strain P. aeruginosa PA14 in close proximity (Fig. 6A), ∼40 nucleotides upstream of our putative TSS (19).

10.1128/mBio.02253-19.7 FIG S6 Expression of the asRhlS. term-seq and strand-specific RNA-seq data (corresponding condition of Fig. 2A) for the *asRhlS* locus in the PAO1 Δ*lasI* Δ*rhlI* (MPK0493), in the AHL^−^ condition. The RNA-seq reads of RhlS (blue line, orange arrow) and asRhlS (red line, red arrow) and the asRhlS term-seq position (green arrow) are indicated. The gap in RNA-seq coverage within *rhlI* is due to the deletion of the ORF. Download FIG S6, PDF file, 0.2 MB. Copyright © 2019 Thomason et al. 2019 Thomason et al. This content is distributed under the terms of the Creative Commons Attribution 4.0 International license.

We could not identify a σ^70^ promoter-like −10 and −35 region for either the putative PAO1 asRhlS +1 or the known PA14 +1, but we did identify a possible promoter consistent with a P. aeruginosa extracytoplasmic function (ECF) σ^E^ consensus (Fig. 6A) upstream of the putative TSS of the asRhlS in PAO1 (40). Surprisingly, disrupting this sequence increased asRhlS expression (Fig. 6B), leading to decreased C4-HSL (Fig. 6C) and about 25% less RhlI protein compared to the wild type (Fig. 4F). P. aeruginosa has 19 ECF sigma factors (reviewed in reference 41). We do not know which of these might be involved in asRhlS induction, and we leave it to future studies to elucidate whether and how asRhlS regulates *rhlI*.

## DISCUSSION

By term-seq mapping of P. aeruginosa TTSs in a QS AHL signal synthesis mutant with or without added AHLs, we identified a number of QS-regulated sRNAs. Our list includes seven AHL-regulated sRNAs, all of which had been detected previously but never reported to be associated with QS (Table 1). Three of these sRNAs were highly induced by AHLs (Table 1, Fig. 1C and D, and Fig. 2A) while the other four showed weaker induction by AHLs. In our previous high-resolution RNA-seq analysis of P. aeruginosa, we described two LasR-activated sRNAs, Lrs1 and Lrs2 (19), which were not identified in our term-seq analysis. We stress that our analysis was not an exhaustive mapping of QS-dependent sRNA expression. We analyzed only one P. aeruginosa strain grown under one condition, 60 min following exposure to AHLs. Furthermore, we used stringent requirements to call a TTS. However, our analysis opens an avenue for future discovery of P. aeruginosa QS-dependent sRNA expression.

Here we focused on the most highly AHL-induced sRNA under our conditions, which we have called RhlS. RhlS is encoded in the 5′ UTR of the C4-HSL synthesis *rhlI* gene, it is 70 nucleotides in length, it appears to require Hfq, it stimulates *rhlI* mRNA translation in a *trans*-acting fashion, and it interferes with the posttranscriptional regulation of an unlinked gene, which codes for the pyoverdine receptor FpvA. Although RhlS was induced when we added both 3OC12-HSL and C4-HSL to growing cells, we presume that induction is primarily a response to 3OC12-HSL because RhlS expression showed a strong dependence on the 3OC12-HSL receptor LasR and only a weak dependence on the C4-HSL receptor RhlR.

The RhlS and *rhlI* transcript start sites appear to be one and the same: There are two RNA isoforms produced from this transcription start site, the shorter RhlS and the longer *rhlS-rhlI* isoforms. Some sRNAs can repress premature transcription termination within a 5′ UTR by binding and inhibiting Rho-dependent termination (42). However, the presence of a Rho-independent terminator at the 3′ end of RhlS (Fig. 3A and 4A) suggests that the long *rhlS-rhlI* isoform might result from leaky or imperfect Rho-independent transcription termination rather than by inhibition of Rho-dependent termination. Whether the efficiency of *rhlS-rhlI* transcription termination changes upon different growth or stress conditions, as described previously for the E. coli SgrS and RybB sRNAs (43), is unclear at this point. However, similar instances of transcriptional readthrough have been shown in other bacteria. For example, in *Salmonella*, leaky transcriptional readthrough of the *IrsK* sRNA terminator leads to a long *lrsK-orf45-anrP* transcript and a stable short *IrsK* sRNA, which can then act in *trans* to increase translation of *orf45* and *anrP* (44). Similarly, incomplete transcription termination of the *Salmonella gltIJKL* operon, which codes for a glutamate-aspartate transporter, produces either the long *gltIJKL* mRNA or a short *gltI* mRNA from which the Hfq-dependent SroC sRNA is processed (45). The SroC sRNA then acts as a sponge to relieve repression of the *gltIJKL* operon. For reasons discussed below, we hypothesize that RhlS may be functioning to relieve interference with *rhlI* mRNA translation perhaps by titration of the asRhlS by a SroC sponge-like mechanism.

We find it interesting that the RhlS isoform but not the full-length isoform required Hfq for their function. It is not surprising that the long isoform does not require Hfq, as *rhlI* transcript levels are minimally altered in an Hfq mutant (17). This sort of differential response of an sRNA and a longer RNA containing the sRNA sequence to Hfq is not unique to RhlS and *rhlI*. Recently it was shown in E. coli the Rho-independent terminators in the Hfq dependent sRNAs SgrS and RyhB allowed transcriptional readthrough, which produces longer Hfq-independent transcripts (43).

Previous work showed that despite minimal changes in the *rhlI* mRNA in the absence of Hfq, translation of *rhlI* and C4-HSL production were reduced in an Hfq mutant (17). We can now explain these reductions. We show that Hfq is important for RhlS, and RhlS is required for normal *rhlI* translation and thus C4-HSL production. We note that regulation of *rhlI* is complex and also affected by sRNAs other than RhlS. The RNA binding protein RsmA, which binds GGA motifs in the loops of RNA hairpins to repress translation, was shown to repress *rhlI* translation and C4-HSL production (46). Additionally, the sRNA RsmY, which binds to RsmA to relive translational repression, has also been implicated in *rhlI* regulation and C4-HSL production by an indirect mechanism involving Hfq stabilization of RsmY (17). Although several GGA motifs are present in RhlS, most are not present in the predicted hairpin loops (although one GGA motif is partially buried), suggesting RhlS likely does not affect translation of *rhlI* through titration of RsmA.

We show that RhlS can function to stimulate *rhlI* translation in a *trans*-acting fashion. RhlS expressed from a multicopy plasmid (at physiologically relevant levels) restores C4-HSL levels almost to those of wild-type PAO1 (Fig. 4E; Fig. S4A). This finding rules out the possibility the regulation is due to inherent factors in the *rhlS-rhlI* transcript itself (e.g., RNA structure alters transcription or translation efficiency of *rhlI*). What is unclear at this point is how RhlS mediates this positive regulation of *rhlI*. We have two models for how this RhlS regulation may occur. First RhlS could act directly on *rhlI* to relieve translational repression mediated by a highly structured 5′ UTR. Mfold predicts the secondary structure of the *rhlI* 5′ UTR occludes the primary RBS by a stem-loop structure (see Fig. S7 in the supplemental material). It is possible RhlS activates *rhlI* in *trans* by base pairing to sequences opposite the *rhlI* RBS, relieving occlusion and facilitating translation. Examples of this type of regulation exist in *Pseudomonas* (and other bacteria) where an sRNA base pairs to a highly structured 5′ UTR to relieve translation repression mediated by RBS occlusion (13, 47–49). Alternatively, as we propose below, RhlS could act in *trans* by a sponge-like mechanism to sequester the asRhlS that appears to repress *rhlI* translation.

10.1128/mBio.02253-19.8 FIG S7 Three structures of the *rhlI* 5′ UTR predicted by Mfold. The sequence spans the *rhlS-rhlI* +1 site of transcription through the first 10 codons of the *rhlI* ORF. The ribosome-binding site for *rhlI* is boxed in blue and is predicted to be occluded within a stem loop in all three structures. Download FIG S7, PDF file, 0.2 MB. Copyright © 2019 Thomason et al. 2019 Thomason et al. This content is distributed under the terms of the Creative Commons Attribution 4.0 International license.

Our term-seq and RNA-seq analyses also uncovered the antisense RNA asRhlS. The existence of this RNA was confirmed by Northern blotting, and the Northern blotting also revealed that while expression of the asRhlS is low compared to that of RhlS, it is detectable in early-logarithmic-phase cells and not in stationary-phase cells (Fig. 6B). The asRhlS overlaps the beginning of the *rhlI* ORF, RBS, and the 5′ UTR of *rhlI.* It is possible that asRhlS base pairs with the *rhlI* mRNA to block translation. Given these data, it is possible that RhlS acts as an asRhlS sponge due to the extensive predicted complementarity between RhlS and asRhlS. Under this hypothesis, this potential interaction between RhlS and asRhlS would sequester asRhlS and relieve the translational repression of *rhlI*. Although this is an intriguing hypothesis, the relationship between RhlS and asRhlS requires further investigation.

Finally, besides having a role in *rhlI* autoregulation, we identified a region of RhlS complementarity in the mRNA of the pyoverdine receptor gene *fpvA.* By analyzing the influence of mutations in RhlS and compensatory mutations in the *fpvA* 5′ UTR on a *fpvA-lacZ* translational fusion we showed that RhlS interferes with translation of *fpvA* via a direct base-pairing mechanism. We have not investigated the physiological significance of this interaction, nor have we searched exhaustively for other potential RhlS-regulated mRNAs; however, we have provided our TargetRNA2 list as a resource for the community (Table S1, tab C). We have thus described an additional layer of gene regulation in the intricate P. aeruginosa quorum sensing circuitry.

## MATERIALS AND METHODS

Details of additional materials and methods are provided in Text S1.

### Bacteria and growth conditions.

The bacterial strains, plasmids, and oligonucleotides used in this study are described in Table S1, tab D. Details of strain construction and experimental growth conditions are listed in the Text S1.

### RNA extraction, library preparation and sequencing.

For RNA-seq, the RNA was extracted using TRIzol and phenol-chloroform. Whole transcriptome RNA-seq libraries and term-seq libraries were prepared as described previously (26).

### RNA extraction for Northern blotting.

RNA extraction for northern analysis was performed by hot acid phenol-chloroform extraction as described previously with minor changes (see Text S1) (50).

### Northern blot analysis.

Northern blotting was performed as described previously (50) with minor modifications. Briefly, RNA was separated on 8% polyacrylamide–6 M urea gel (National Diagnostics) and transferred to a Hybond-XL membrane (GE Healthcare). Membranes were probed with [^32^P]ATP end-labeled oligonucleotides specific to the desired transcript (Table S1, tab D) and exposed to Amersham Hyperfilm MP (GE-Healthcare) at –80°C.

### C4-HSL and β-galactosidase measurements.

C4-HSL was ethyl acetate extracted from 24-h LB plus 50 mM MOPS (morpholinepropanesulfonic acid) culture supernatant as described previously (51). The amount of C4-HSL was determined by using an E. coli (pECP61.5) bioassay (52, 53) and the Tropix Galacto-Light Plus reagent (Invitrogen).

### Data availability.

RNA-seq and term-seq data sets have been deposited in the European Nucleotide Database (ENA) under study accession no. PRJEB31965.
