# Supplementary material for: A rhlI 5′ UTR-Derived sRNA Regulates RhlR-Dependent Quorum Sensing in Pseudomonas aeruginosa
Source: mBio. 2019 Oct 8;10(5):e02253-19. doi: 10.1128/mBio.02253-19 (PMC6786874; doi:10.1128/mBio.02253-19)
Supplement: TEXT S1 [file mBio.02253-19-s0001.pdf]

A *rhlI* 5' UTR-derived sRNA regulates RhlR-dependent quorum sensing in *Pseudomonas aeruginosa*

Maureen K. Thomason, Maya Voichek, Daniel Dar, Victoria Addis, David Fitzgerald, Susan Gottesman, Rotem Sorek and E. Peter Greenberg

TEXT S1: SUPPLEMENTARY MATERIALS AND METHODS

Bacteria and growth conditions. The bacterial strains, plasmids and oligonucleotides used are described in Table S1D. For standard cloning and strain construction, or unless otherwise indicated, bacteria were grown in lysogeny broth (LB-Lennox) (10 g tryptone, 5 g NaCl, 5 g yeast extract per liter) or LB with 50 mM pH 7 MOPS buffer (LB MOPS) at 37°C with shaking at 250 rpm. For *Pseudomonas* cultures unless otherwise indicated overnight cultures were grown in LB + 50 mM MOPS at 37°C were diluted back in fresh LB + 50 mM MOPS, grown to early exponential phase (OD_{600} ~ 0.2-0.3) and were diluted in fresh medium to an OD_{600} of ~0.005. Samples were collected at the indicated times and/or OD600 values indicated in the figures. Where indicated the appropriate AHLs were added at the following final concentrations: C4-HSL (Cayman Chemicals) at 10 µM and 3OC12-HSL (RTI International) at 2 µM. Antibiotics were used at the following concentrations as needed: for *Pseudomonas* gentamicin 100 µg/ml, carbenicillin 300 µg/mL, and tetracycline 100 µg/mL, and for *E. coli* gentamicin 10 µg/ml and ampicillin 100 µg/mL.

Strain construction. Deletion and point mutant strains were constructed by homologous recombination as follows: (1) ~500nt of upstream or downstream DNA homology regions of the relevant gene to be deleted were amplified by overlapping PCR using the primers listed in Table S1D, cloned into pEXG2 (2) and mobilized from *E. coli* strain S17-1 (3) into *P. aeruginosa* PAO1 by conjugation.

For the RhlS-Δ48-71 term mutant the upstream flanking PCR was generated using primers MT0231 and MT0232. The downstream flanking region (including the Δ48-71 term mutation) was constructed using primers MT0233 that contains homology to MT0232 and deletes residues 48-71 of RhlS and MT0234. The final overlapping PCR to create the RhlS-Δ48-71 term mutant with the appropriate flanking DNA sequence was generated using primers MT0235 and MT0236 that contained PstI and XhoI sites for cloning into the pEXG2 integration vector. For the MutA and MutA+B point mutant strains the flanking PCR was generated as described above for the RhlS-Δ48-71 term mutant with the following modifications. For the MutA mutant, primer MT0278 (containing the G61C, C62A, C63A, U64G nucleotide changes) was used with primer MT0231 to generate the upstream homology region including the nucleotide changes while MT0279 and MT0234 were used to generate the downstream homology region. For the MutA+B mutant the upstream homology region was amplified using primers MT0322 (containing the G48C, G49T, G50T, U51G, G61C, C62A, C63A, U64G nucleotide changes) with primer MT0231. The downstream homology region was amplified with MT0323 and MT0234. PCR for the asRhlS-1 mutant was performed in three stages. Primers MT0290 and MT0291 were used first to generate upstream homology to the asRhlS locus. A second PCR was performed with primers MT0299 and MT0293 to generate the C-7G, C-10G, T-13G, G-34T nucleotide changes (positions relative to putative +1 of asRhlS transcription). The final overlapping PCR was performed with primers MT0294 and MT0295 that contain HindIII and SacI sites for cloning into pEXG2 plasmid. All plasmid inserts were verified by sequencing using primers MK0018 and MK0019.

Upon conjugation into *Pseudomonas*, transconjugants were selected on *Pseudomonas* Isolation Agar (Difco) with 100 μg/ml gentamicin. Unmarked deletion or point mutation strains were created by counter selection on LB (lacking NaCl) with 10% sucrose. The MPK0493 deletion strain is deleted for most of the ORF, except the firsat 100 nts and the last 54 nts, with 5

additional nts, retaining the reading frame. The *fpvA-lacZ* (and mutant) translational fusions were constructed as described previously (4) by fusing the 5' UTR through the 25th codon of *fpvA* in frame to *lacZ* in *E. coli* strain PM1205. The *fpvA-1-lacZ* mutant was constructed by using overlapping PCR to introduce the point mutations as described previously (1). To construct the P_{BAD}-*rhlS-rhlI-lacZ* fusion the entire 5' UTR encompassing RhlS and the first 30 codons of *rhlI* was amplified and recombineered in frame at the *lacZ* locus in *E. coli* PM1205. To introduce the P_{BAD}-*rhlS-rhlI* ORF into *E. coli* the 5' UTR including all of RhlS and the entire *rhlI* open reading frame was amplified and recombineered into the *lac* locus, replacing *lacZ*. All strains and point mutations were verified by PCR and DNA sequencing.

Plasmid construction*.* To construct the pMKT1 sRNA expression vector, the *araC-P_{BAD}-MCS-rrnB* fragment from the *E. coli* sRNA expression plasmid pEF21 (5) was amplified by overlapping PCR as follows (sequence of the oligonucleotides are listed in Table S1D). The *araC* fragment was amplified from pEF21 with primers MT0245 and MT0246 which eliminates the SapI site. A second PCR reaction to amplify the Pbad promoter cassette with homology to the *araC* fragment was constructed using MT0247 and MT0248, this replaces the PstI site with a SpeI site for sRNA cloning. The *araC-Pbad* cassette was constructed by overlapping PCR using MT0245 and MT0248. The MCS-rrnB fragment was generated using primers MT0249 and MT0250 that contain homology to the *araC-Pbad* fragment. Overlapping PCR was used to generate the *araC-Pbad-MCS-rrnB* fragment using *araC-Pbad* and *MCS-rrnB* fragments templates with primers MT0251 and MT0252. The resulting fragment was cloned into the SapI and SfiL sites of pCUP22 (6) and verified by sequencing using primers MT0259-MT0264 to tile across the entire insert. The resulting plasmid was named pMKT1.

For arabinose inducible expression of pRhlS or pMutC from the pMKT1 plasmid, the corresponding RhlS fragment was amplified and cloned into pMKT1 plasmid. For RhlS primers MT0308 and MT0289 were used to amplify RhlS with SpeI and HindIII sites for cloning. For the

MutC mutant the fragment was PCR amplified using primers MT0308 and MT0383 (containing the C10G, U11A, G12C, G13C point mutations) and cloned into the SpeI and HindIII sites of pMKT1. All plasmid inserts were verified by DNA sequencing using primers MT0309 and MT0310.

RNA extraction for RNA-seq and term-seq. To obtain total RNA samples for sequencing, overnight cultures of the *P. aeruginosa ΔlasI, ΔrhlI* mutant (MPK0493) grown in LB MOPS were used as inocula. Cells were grown in LB MOPS (starting OD_{600} of 0.01) and when the culture density reached an OD_{600} of 0.2-0.3 cells were reinoculated into LB MOPS to a starting density of 0.005. When these cultures reached an OD_{600} of 0.8 they were split in two, and C4-HSL, 10 µM and 3-oxo-C12-HSL, 2 µM were both added to one of the culture flasks. The other culture was left as an untreated control. After 60 min incubation cells from 10 mL of AHL treated and untreated cultures were incubated with 2 mL stop solution (95% Ethanol, 5% acid phenol, pH 4.5, Ambion) and placed on ice for 10 min. The chilled cells were pelleted by centrifugation, and the pellets were snap frozen and stored at -80°C. RNA was extracted as follows: Cell pellets were suspended in 100 µL of 10 mg/mL lysozyme in 10 mM Tris, 1mM EDTA, pH 8.0, and incubated at 37°C for 2 min with occasional gentle mixing. One mL of Trizol reagent (Invitrogen) was added, samples were vortexed vigorously, and transferred to Phase Lock Gel 2.0 tubes (5Prime) containing 500 µL chloroform. After an additional incubation at room temperature for 5 min the supernatant fluid was again clarified by microcentrifugation at 4°C for 15 min. The clarified supernatant fluid was combined with 600 µl phenol-chloroform-IAA (Invitrogen) in a new Phase Lock Gel 2.0 tube, mixed by inversion and and clarified by centrifuged. This clarified supernatant fluid was combined with 600 µl isopropanol, vortexed and after 10 min at room temperature it was stored at -20°C overnight. The RNA was then precipitated, washed with 70% ethanol and suspended in DEPC-H_2O. RNA concentration was determined by absorbance at OD_{260}, in addition to using Qubit® RNA BR Assay Kit (Life technologies, Q10210). RNA integrity

was checked with Tapestation (Agilent, 5067-5576). All RNA samples were treated with TURBO™ DNase I following manufacturer's instructions (Life technologies, AM2238). The absence of genomic DNA contamination was confirmed by PCR with the oligonucleotides MK0054 and MK0055.

RNA-seq and term-seq library preparation and sequencing. Strand specific whole transcriptome RNA libraries and term-seq libraries were prepared as described previously (7). All libraries were sequenced on Illumina Miseq platform. Sequenced reads were de-multiplexed and adapters were trimmed by using Casava v1.8.2. (Illumina).

Read mapping and data analysis*.* Reads were mapped to the *P. aeruginosa* PAO1 reference genome (Genbank: NC_002516) using NovoAlign V3.02.02 (Novocraft) with default parameters, as described previously (7). For RNA-seq, the mapped reads were used to generate genome-wide coverage maps. Term-seq positions were determined as the first nucleotide position of the mapped read, and their counts were calculated per nucleotide position in the genome as described in (7).

Transcription termination site (TTS) mapping. Term-seq sites that were <10 nucleotides apart were filtered, leaving the site with most counts as the "dominant site". Dominant sites located less than 150 nucleotides downstream of a gene were associated with the gene (Table S1A). Genes that had several dominant sites were further filtered and the term-seq site was chosen manually in accordance with whole transcriptome RNA-seq coverage. Term-seq sites that were associated with multiple short consecutive genes in this analysis (e.g. tRNAs) were manually examined and only associated with the correct corresponding upstream gene. All dominant gene-associated term-seq ends identified in the 10' –AHLs condition are listed in Table S1A.

Reproducible term-seq sites that had at least 2 reads in each of the three biological replicates were analyzed against the term-seq sites found in the contrasting condition (-AHLs vs. +AHLs in

the 60' time point) in the following manner: All reproducible sites that were <10 nucleotides away from each other and on the same strand were clustered together, and the total sum of reads for that cluster was assigned for each biological replicate. The term-seq reads corresponding to the same positions in the cluster from the contrasting condition were added up similarly. Then, a differential expression analysis for all clusters was carried out using the R package DESeq2 (8), with the raw read counts from the 3 sets of biological replicates for each condition as input, and accounting for batch effect. A significant adjusted p-value (FDR<0.1) was considered as the threshold for differentially expressed term-seq sites. For each cluster, the dominant term-seq site per condition (the one supported by maximum normalized reads) was presented in Table S1B, with the average number of normalized reads from the 3 replicates indicated.

RNA extraction for northern blotting and RACE. RNA extraction for northern analysis and RACE was performed by using hot acid phenol chloroform (pH 4.5, Ambion) as described previously with minor changes (9). Cell pellets for RNA extraction were thawed, resuspended in 100 µL of lysozyme buffer (10 mg/mL lysozyme in 10 mM Tris, 1mM EDTA, pH 8.0) and incubated at 37°C for 2 min with occasional gentle mixing. Lysates were combined with 500 µl DEPC-water. RNA was then extracted, pelleted, washed and resuspended as described (9). RNA concentration was measured on a Nanodrop by reading the absorbance at OD_{260}.

Northern blot and RACE analysis. Northern blotting was performed as described previously (9) with minor modifications. Briefly, RNA was separated on 8% polyacrylamide-6M urea gel (National Diagnostics) and transferred to a Hybond-XL membrane (GE-Healthcare). To determine RNA sizes, $^{\alpha\text{-}32P}$-ATP was used to radiolabel by *in vitro* transcription of the RNA Century Marker Plus Template (Ambion) to produce an RNA marker of known size. The position of the sizes indicated on the figures corresponds to the appropriately sized RNA band.

Membranes were probed with ^{32P}-ATP end-labeled oligonucleotides specific to the desired transcript (Table S1D) and exposed to Amersham Hyperfilm MP (GE-Healthcare) at -80°C.

RACE end mapping was performed using the oligonucleotides listed in Table S1D as described previously (10) with minor modifications. To remove the 5' PPP from RNA, 15 µg total RNA was incubated with RppH (20U, NEB) in place of the no longer available Tobacco Acid Pyrophosphatase (TAP) at 37°C for 1h. cDNA products were cloned into pCR2.1-TOPO cloning vector (Invitrogen) and sequenced.

Primer extension analysis. RNA was extracted from *P. aeruginosa* PAO1 as described previously (11). To detect the 5' end of RhlS, primer VA0001 (corresponding to nucleotides -61 to -42 relative to the *rhlI* start of translation) was end-labeled with [γ-^{32}P] ATP (MP Biochemicals-ICN) using T4 polynucleotide kinase (NEB) for 1 h at 37°C, purified and then added to 5 µg of total RNA. The reaction mixture was incubated at 95°C for 5 min, slow cooled to 42°C, and then incubated for 1 h with 0.5 µL Superscript III RNase H- reverse transcriptase (Invitrogen), 2 µl of 5X First-Strand buffer, 0.5 µl 0.1M DTT, and 1 µl 10mM dNTPs. Reactions were terminated by adding 7 µl of Stop buffer from a SequiTherm EXCEL II DNA Sequencing Kit (Epicentre Biotechnologies) and stored at -20°C. To generate a template for the sequencing ladder, *rhlI* and *rhlR* were amplified from *P. aeruginosa* PAO1 chromosomal DNA with primers VA0002 and VA0003, gel extracted and cloned into the pCR2.1 TOPO cloning vector (Invitrogen). We used this plasmid as a template to generate the sequencing ladder with the SequiTherm EXCEL II DNA Sequencing Kit (Epicentre Biotechnologies). To resolve the 5' end of RhlS, primer extension reactions and the DNA sequencing ladder were subjected to gel electrophoresis on an 8% polyacrylamide-6M urea gel (National Diagnostics) and exposed to KODAK BioMax XAR film at -80°C.

C4-HSL and b-galactosidase measurements. C4-HSL was ethyl acetate extracted from 24 h LB MOPS culture fluid as described previously (12). The amount of C4-HSL was determined by

using an *E. coli* (pECP61.5) bioassay (13, 14) and the Tropix Galacto-Light Plus reagent (Invitrogen). To detect RhlS-*fpvA* base pairing and disruption we measured β-galactosidase levels 3 hours after induction with 0.4% arabinose as described previously (9).

qRT-PCR. To generate cDNA we used 1 μg of total RNA (free of gDNA contamination) in the iScript Select cDNA Synthesis kit (Bio-Rad) with random primers according the manufacturer's instructions. The total amount of mRNA for each gene was determined by using SYBR Green PCR MasterMix (Applied Biosystems/Thermo) and gene specific primers listed in Table S1D. qRT-PCR was performed on a CFX Real Time Machine (Bio-Rad). In each case, the total amount of mRNA was determined by using a standard curve and normalizing to levels of the *groEL* mRNA.

Western blotting. Western blot analysis was performed as described previously (15) with the following modifications. Cells were grown in LB MOPS at 37°C for 24 h. Polypeptides were separated on a 12% NuPAGE Novex Bis-Tris Protein Gel (Life Technolgies) and transferred to a nitrocellulose membrane (Invitrogen). The membrane was blocked in Odyssey Blocking PBS Buffer (Li-Cor Biosciences) and probed with 1:5000 dilution of α-VSV-G antibody (Sigma) or 1:5000 dilution of α-RNAP antibody (Neoclone Biotechnology). For the RhlI western, the membrane was subsequently probed with IRDye 800CW Goat anti-Rabbit IgG (Li-Cor Biosciences), and for the RNAP western the membrane was probed with IRDye 680LT Goat anti-Mouse IgG (Li-Cor Biosciences). Membranes were washed in 1X PBS + 0.1% Tween-20 and scanned using an Odyssey CLx Imaging System (Li-Cor Biosciences). Intensities of the RhlI bands were quantified and normalized to the corresponding RNAP bands using ImageJ (16).

Data availability. RNA-seq and term-seq datasets were deposited in the European Nucleotide Database (ENA), study accession no. PRJEB31965.

REFERENCES FOR TEXT S1: SUPPLEMENTARY MATERIALS AND METHODS

1. Ho SN, Hunt HD, Horton RM, Pullen JK, Pease LR. 1989. Site-directed mutagenesis by overlap extension using the polymerase chain reaction. Gene 77:51-59.
2. Rietsch A, Mekalanos JJ. 2006. Metabolic regulation of type III secretion gene expression in *Pseudomonas aeruginosa*. Mol Microbiol 59:807-820.
3. Simon R, Priefer U, Pühler A. 1983. A broad host range mobilization system for in vivo genetic engineering: transposon mutagenesis in Gram negative bacteria. Nature Biotechnology 1:784-791.
4. Mandin P, Gottesman S. 2009. A genetic approach for finding small RNAs regulators of genes of interest identifies RybC as regulating the DpiA/DpiB two-component system. Mol Microbiol 72:551-565.
5. Fozo EM, Kawano M, Fontaine F, Kaya Y, Mendieta KS, Jones KL, Ocampo A, Rudd KE, Storz G. 2008. Repression of small toxic protein synthesis by the Sib and OhsC small RNAs. Mol Microbiol 70:1076-1093.
6. West SE, Schweizer HP, Dall C, Sample AK, Runyen-Janecky LJ. 1994. Construction of improved *Escherichia-Pseudomonas* shuttle vectors derived from pUC18/19 and sequence of the region required for their replication in *Pseudomonas aeruginosa*. Gene 148:81-86.
7. Dar D, Shamir M, Mellin JR, Koutero M, Stern-Ginossar N, Cossart P, Sorek R. 2016. Term-seq reveals abundant ribo-regulation of antibiotics resistance in bacteria. Science 352:aad9822.

8. Love MI, Huber W, Anders S. 2014. Moderated estimation of fold change and dispersion for RNA-seq data with DESeq2. Genome Biol 15:550.

9. Thomason MK, Fontaine F, De Lay N, Storz G. 2012. A small RNA that regulates motility and biofilm formation in response to changes in nutrient availability in *Escherichia coli*. Mol Microbiol 84:17-35.

10. Argaman L, Hershberg R, Vogel J, Bejerano G, Wagner EG, Margalit H, Altuvia S. 2001. Novel small RNA-encoding genes in the intergenic regions of *Escherichia coli*. Curr Biol 11:941-950.

11. Wilderman PJ, Sowa NA, FitzGerald DJ, FitzGerald PC, Gottesman S, Ochsner UA, Vasil ML. 2004. Identification of tandem duplicate regulatory small RNAs in *Pseudomonas aeruginosa* involved in iron homeostasis. Proc Natl Acad Sci U S A 101:9792-9797.

12. Pearson JP, Gray KM, Passador L, Tucker KD, Eberhard A, Iglewski BH, Greenberg EP. 1994. Structure of the autoinducer required for expression of *Pseudomonas aeruginosa* virulence genes. Proc Natl Acad Sci U S A 91:197-201.

13. Parsek MR, Schaefer AL, Greenberg EP. 1997. Analysis of random and site-directed mutations in *rhlI*, a *Pseudomonas aeruginosa* gene encoding an acylhomoserine lactone synthase. Mol Microbiol 26:301-310.

14. Pearson JP, Pesci EC, Iglewski BH. 1997. Roles of *Pseudomonas aeruginosa las* and *rhl* quorum-sensing systems in control of elastase and rhamnolipid biosynthesis genes. J Bacteriol 179:5756-5767.

15. Hemm MR, Paul BJ, Schneider TD, Storz G, Rudd KE. 2008. Small membrane proteins found by comparative genomics and ribosome binding site models. Mol Microbiol 70:1487-1501.

16. Schneider CA, Rasband WS, Eliceiri KW. 2012. NIH Image to ImageJ: 25 years of image analysis. Nat Methods 9:671-675.
