# Supplementary material for: A rhlI 5′ UTR-Derived sRNA Regulates RhlR-Dependent Quorum Sensing in Pseudomonas aeruginosa
Source: mBio. 2019 Oct 8;10(5):e02253-19. doi: 10.1128/mBio.02253-19 (PMC6786874; doi:10.1128/mBio.02253-19)
Supplement: FIG S1 [file mBio.02253-19-sf001.pdf]

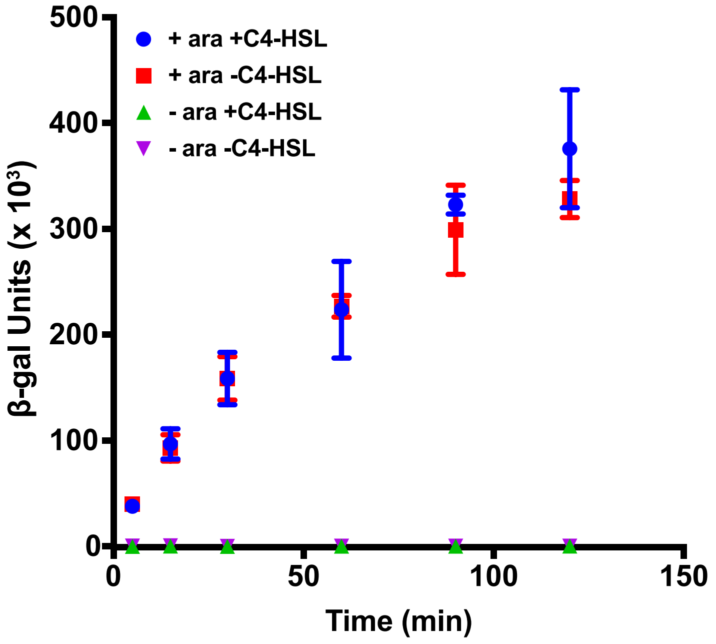

Figure S1: The 5' UTR of *rhlI* does not respond to exogenous C4-HSL as a riboswitch. Overnight cultures of *E. coli* P_{BAD}-*rhlS-rhlI::lacZ* (MPK0601) were diluted into 100 mL fresh LB+50 mM MOPS in 500-mL baffled flasks and grown at 37°C with shaking. At an OD_{600} ~0.4 the cultures were split and either only 10 µM C4-HSL, only 0.2% arabinose, both or neither were added to each flask. At the indicated times, samples were collected and β-galactosidase levels were determined.
