# Supplementary material for: A rhlI 5′ UTR-Derived sRNA Regulates RhlR-Dependent Quorum Sensing in Pseudomonas aeruginosa
Source: mBio. 2019 Oct 8;10(5):e02253-19. doi: 10.1128/mBio.02253-19 (PMC6786874; doi:10.1128/mBio.02253-19)
Supplement: FIG S2 [file mBio.02253-19-sf002.pdf]

(A)

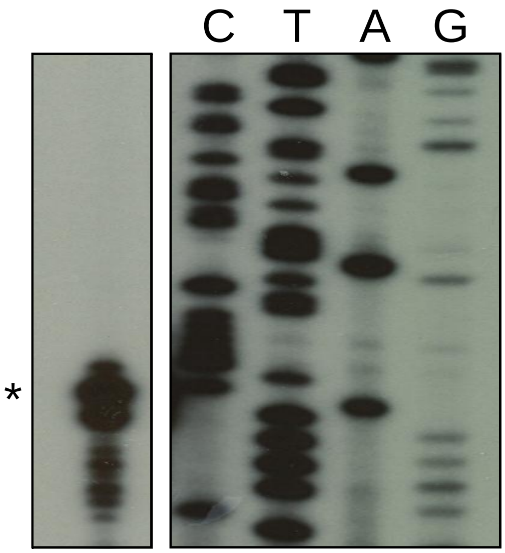

(B)

Gen	PCR Primer	RppH[a]	Clone[b]	Sequence
RhlS	MT0232	+	1-1	ATGTGTGTGCTGGTATGTCCTCCGACTGAGAGGGCCCAGGAGTATCA
RhlS	MT0232	+	1-2	GCTGGTATGTCCTCCGACTGAGAGGGCCCAGGAGTATCA
RhlS	MT0232	+	1-3	ATGTGTGTGCTGGTATGTCCTCCGACTGAGAGGGCCCAGGAGTATCA
RhlS	MT0232	+	1-4	ATGTGTGTGCTGGTATGTCCTCCGACTGAGAGGGCCCAGGAGTATCA
RhlS	MT0232	+	1-5	ATGTGTGTGCTGGTATGTCCTCCGACTGAGAGGGCCCAGGAGTATCA
RhlS	MT0232	+	1-6	ATGTGTGTGCTGGTATGTCCTCCGACTGAGAGGGCCCAGGAGTATCA
RhlS	MT0232	+	1-7	ATGTGTGTGCTGGTATGTCCTCCGACTGAGAGGGCCCAGGAGTATCA
RhlS	MT0232	+	1-8	ATGTGTGTGCTGGTATGTCCTCCGACTGAGAGGGCCCAGGAGTATCA
RhlS	MT0232	+	1-9	ATGTGTGTGCTGGTATGTCCTCCGACTGAGAGGGCCCAGGAGTATCA
RhlS	MT0232	+	2-3	ATGTGTGTGCTGGTATGTCCTCCGACTGAGAGGGCCCAGGAGTATCA
RhlS	MT0232	+	2-4	TCATGTGTGTGCTGGTATGTCCTCCGACTGAGAGGGCCCAGGAGTATCA
RhlS	MT0232	+	2-5	ATGTGTGTGCTGGTATGTCCTCCGACTGAGAGGGCCCAGGAGTATCA
RhlS	MT0232	+	2-6	ATGTGTGTGCTGGTATGTCCTCCGACTGAGAGGGCCCAGGAGTATCA
RhlS	MT0232	+	2-8	ATGTGTGTGCTGGTATGTCCTCCGACTGAGAGGGCCCAGGAGTATCA
RhlS	MT0232	+	2-9	ATGTGTGTGCTGGTATGTCCTCCGACTGAGAGGGCCCAGGAGTATCA
RhlS	MT0232	+	2-10	ATGTGTGTGCTGGTATGTCCTCCGACTGAGAGGGCCCAGGAGTATCA
RhlS	MT0232	+	2-11	ATGTGTGTGCTGGTATGTCCTCCGACTGAGAGGGCCCAGGAGTATCA
RhlS	MT0232	+	2-12	ATGTGTGTGCTGGTATGTCCTCCGACTGAGAGGGCCCAGGAGTATCA
RhlS	MT0232	-	7-1	ATGTGTGGGCTGGTATGTCCTCCGACTGAGAGGGCCCAGGAGTATCA
RhlS	MT0232	-	7-2	ATGTGTGTGCTGGTATGTCCTCCGACTGAGAGGGCCCAGGAGTATCA
RhlS	MT0232	-	7-3	ATGTGTGTGCTGGTATGTCCTCCGACTGAGAGGGCCCAGGAGTATCA
RhlS	MT0232	-	7-4	ATGTGTGTGCTGGTATGTCCTCCGACTGAGAGGGCCCAGGAGTATCA
RhlS	MT0232	-	8-1	TGCTGGTATGTCCTCCGACTGAGAGGGCCCAGGAGTATCA
RhlS	MT0232	-	8-3	TCATGTGTGTGCTGGTATGTCCTCCGACTGAGAGGGCCCAGGAGTATCA
RhlS	MT0232	-	8-4	ATGTGTGTGCTGGTATGTCCTCCGACTGAGAGGGCCCAGGAGTATCA
rhlI	MT0238		3-3	ATGTGTGTGCTGGTATGTCCTCCGACTGAGAGGGCCCAGGAGTATCAGGGTAGGGATGC<br>CGCCTTTTTTTTCTCGGCCGGCACGACACGGGGACTTGGTCATGATCGAATTGCTCTCTGAATCGCTGGAAGGGCTTT
rhlI	MT0238		3-4	ATGTGTGTGCTGGTATGTCCTCCGACTGAGAGGGCCCAGGAGTATCAGGGTAGGGATGC<br>CGCCTTTTTTTTCTCGGCCGGCACGACACGGGGACTTGGTCATGATCGAATTGCTCTCTGAATCGCTGGAAGGGCTTT
rhlI	MT0238		4-11	GACTTGGTCATGATCGAATTGCTCTCTGAATCGCTGGAAGGGCTTT
rhlI	MT0238		4-13	ATGATCGAATTGCTCTCTGAATCGCTGGAAGGGCTTTCC
rhlI	MT0347		5-8	ATGTGTGTGCTGGTATGTCCTCCGACTGAGAGGGCCCAGGAGTATCAGGGTAGGGATGC<br>CGCCTTTTTTTTCTCGGCCGGCACGACAC

[a]Used to determine if a transcript 5' end is a primary transcription start or processed transcription start. No difference in +/- RppH (RNA 5' Pyrophosphohydrolase) treatment indicates the 5' end of both RhlS and *rhlI* are primary transcription start sites.
[b]Number of pCRII-TOPO clone. Each independent clone indicates a separate RNA transcript that was sequenced

Figure S2: Analysis of transcription start sites identifies a single transcription start for RhlS and *rhlI*. (A) Primer extension analysis. To detect the 5’ end of RhlS, primer VA0001 (corresponding to nt -61 to -42 relative to the *rhlI* start of translation) was end-labeled with [γ-^{32}P] ATP and incubated with the primer extension enzyme mix. The *rhlI*-*rhlR* fragment was amplified from PAO1 to generate the sequencing ladder. To resolve the 5’ end of RhlS, primer extension reactions and the DNA sequencing ladder were run on an 8% polyacrylamide-6M urea gel. *Corresponds to +1 of RhlS indicated in Fig 3A. (B) 5' RACE data for RhlS and *rhlI*. Total RNA isolated from WT PAO1 was subjected to 5’ RACE analysis using the indicated primers. Each row represents an independent clone (and RNA transcript) that was sequenced.
