# Supplementary material for: A rhlI 5′ UTR-Derived sRNA Regulates RhlR-Dependent Quorum Sensing in Pseudomonas aeruginosa
Source: mBio. 2019 Oct 8;10(5):e02253-19. doi: 10.1128/mBio.02253-19 (PMC6786874; doi:10.1128/mBio.02253-19)
Supplement: FIG S3 [file mBio.02253-19-sf003.pdf]

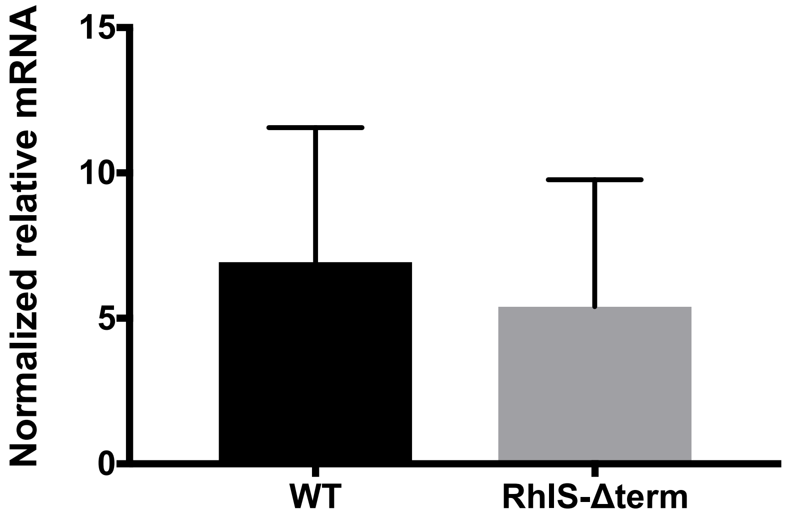

Figure S3: Levels of the *rhlI* mRNA are similar in the WT and the RhlS-Δterm mutant. Single colonies of WT PAO1 and the RhlS-Δterm mutant (MPK0555) were grown for 24 h in 10mL LB+50 mM MOPS in 50 mL flasks at 37°C with shaking. Total RNA was extracted, cDNA was generated and mRNA levels were measured as in Fig 4C. Results are means of two independent experiments with two technical replicates each and error bars are standard deviations.
