# Supplementary material for: A rhlI 5′ UTR-Derived sRNA Regulates RhlR-Dependent Quorum Sensing in Pseudomonas aeruginosa
Source: mBio. 2019 Oct 8;10(5):e02253-19. doi: 10.1128/mBio.02253-19 (PMC6786874; doi:10.1128/mBio.02253-19)
Supplement: FIG S4 [file mBio.02253-19-sf004.pdf]

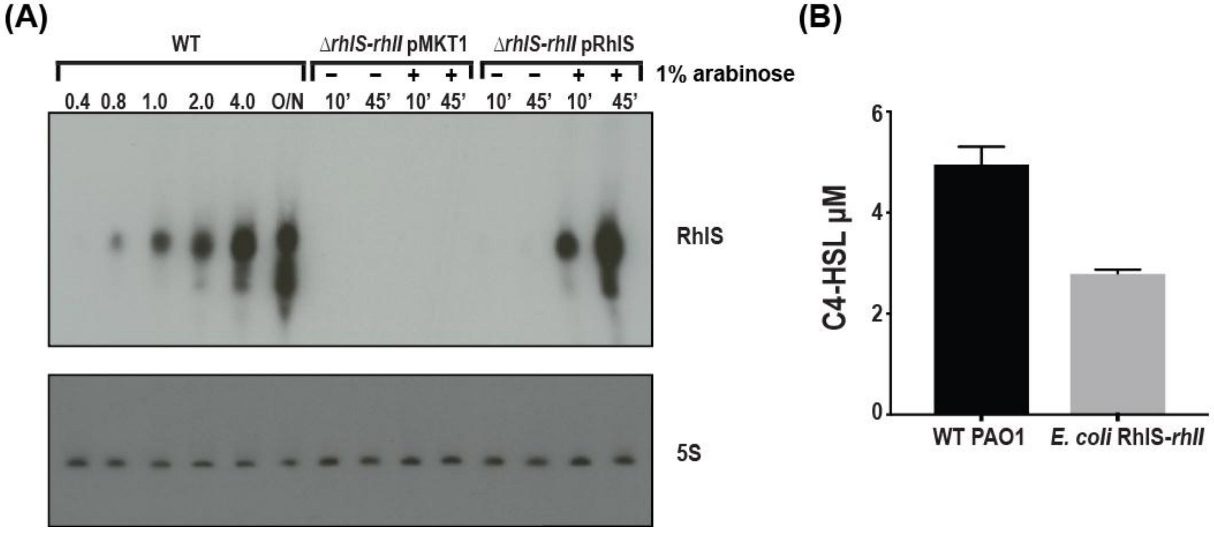

Figure S4: Ectopic expression of RhlS is physiologically relevant. (A) RhlS levels in the *rhlS-rhlI* mutant (MPK0627) containing pRhlS or the vector pMKT1 are similar to WT PAO1. For the mutants, overnight cultures were diluted to OD_{600} ~0.005 in LB + 50 mM MOPS and at OD_{600} ~0.4 the cultures were split and either no or 1% L-arabinose was added. Samples were collected at the indicated times after addition of arabinose. For wild type PAO1 cells were grown similarly but arabinose was not added and culture densities at sampling times are shown. For all samples, RNA was extracted and analyzed by northern analysis as in Fig. 2B. (B) Levels of C4-HSL produced by *E. coli* with a chromosomal insertion of *rhl* compared to levels produced by wild type *P. aeruginosa* PAO1. Single colonies of PAO1 or *E. coli* PM1205-P_{BAD}-*rhlS-rhlI* (MPK0603) were used to inoculate 10 mL LB + 50mM MOPS in 50 mL flasks and grown at 37°C in the presence of 1% L-arabinose for 18 h, C4-HSL was extracted and measured as in Fig. 4B. Data are the means of three biological replicates and error bars are standard deviations.
