# Supplementary material for: A rhlI 5′ UTR-Derived sRNA Regulates RhlR-Dependent Quorum Sensing in Pseudomonas aeruginosa
Source: mBio. 2019 Oct 8;10(5):e02253-19. doi: 10.1128/mBio.02253-19 (PMC6786874; doi:10.1128/mBio.02253-19)
Supplement: FIG S5 [file mBio.02253-19-sf005.pdf]

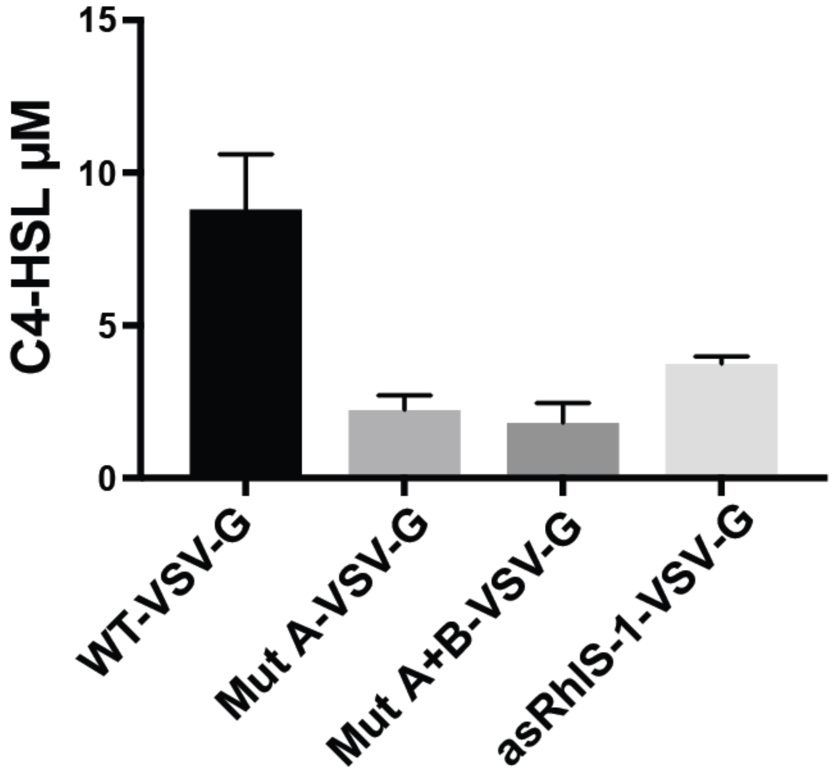

Figure S5: The RhlI-VSV-G epitope tag does not alter C4-HSL production. Single colonies of WT RhlI-VSV-G (MPK0698), RhlS Mut A-VSV-G (MPK0689), RhlS Mut A+B VSV-G (MPK0697) and the asRhlS-1-VSV-G (MPK0687) strains were grown for 24 h in 10 mL LB + 50mM MOPS in 50 mL flasks at 37°C with shaking. C4-HSL was measured as described in Fig 4B. Data are from three biological and two technical replicates for each strain and error bars are standard deviations.
