# Supplementary material for: A rhlI 5′ UTR-Derived sRNA Regulates RhlR-Dependent Quorum Sensing in Pseudomonas aeruginosa
Source: mBio. 2019 Oct 8;10(5):e02253-19. doi: 10.1128/mBio.02253-19 (PMC6786874; doi:10.1128/mBio.02253-19)
Supplement: FIG S6 [file mBio.02253-19-sf006.pdf]

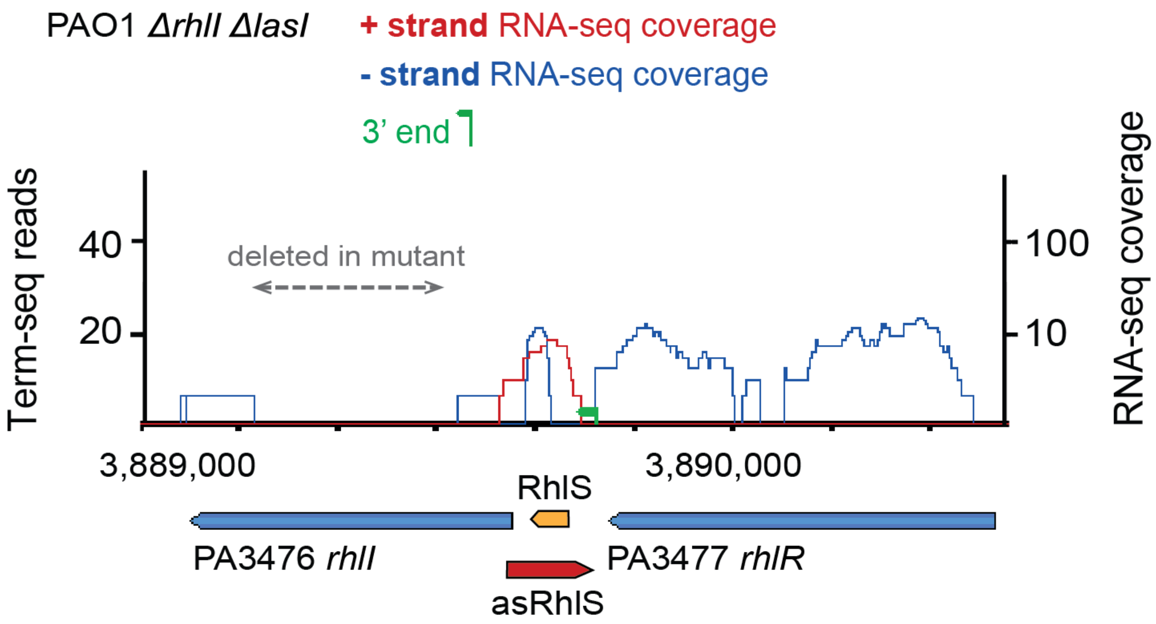

Figure S6: Expression of the asRhlS. Term-seq and strand-specific RNA-seq data (corresponding condition of Fig 2A) for the *asRhlS* locus in the PAO1 Δ*lasI*Δ*rhlI* (MPK0493), in the –AHLs condition. The RNA-seq reads of RhlS (blue line, orange arrow), asRhlS (red line, red arrow) and the asRhlS term-seq position (green arrow) is indicated. The gap in RNA-seq coverage within *rhlI* is due to the deletion of the ORF.
