# Supplementary material for: A rhlI 5′ UTR-Derived sRNA Regulates RhlR-Dependent Quorum Sensing in Pseudomonas aeruginosa
Source: mBio. 2019 Oct 8;10(5):e02253-19. doi: 10.1128/mBio.02253-19 (PMC6786874; doi:10.1128/mBio.02253-19)
Supplement: FIG S7 [file mBio.02253-19-sf007.pdf]

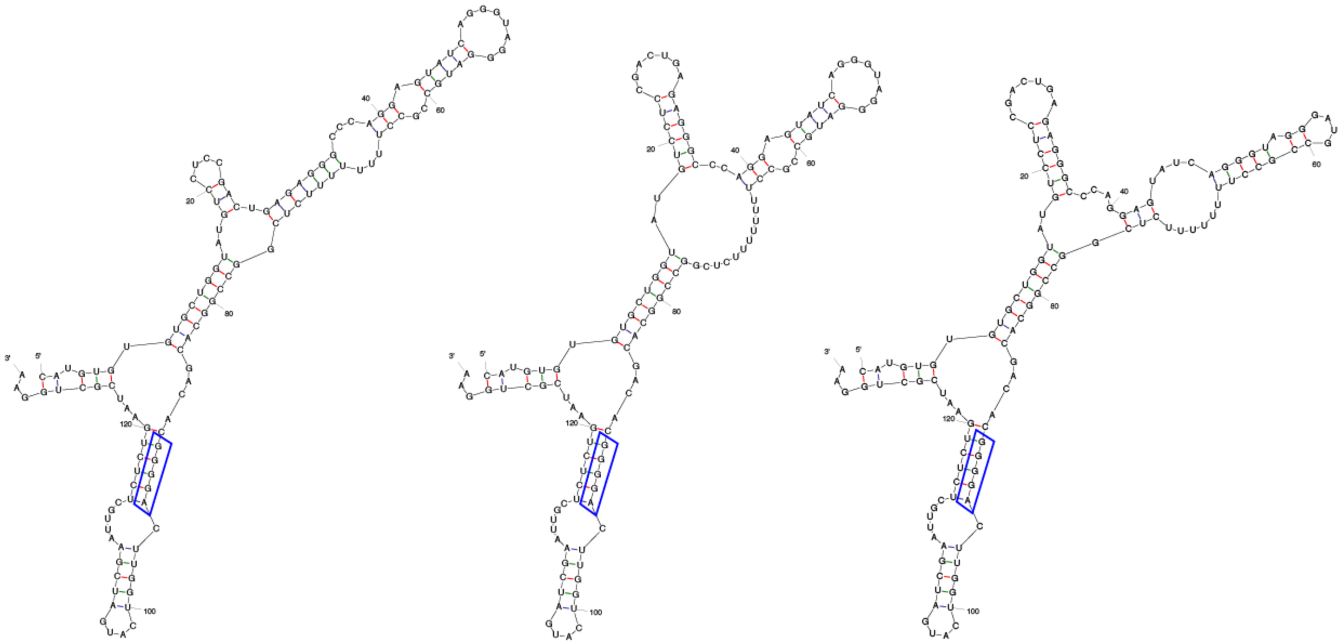

Figure S7. Three structures of the *rhlI* 5' UTR predicted by Mfold. The sequence spans the *rhlS-rhlI* +1 of transcription through the first 10 codons of the *rhlI* ORF. The ribosome-binding site for *rhlI* is boxed in blue and is predicted to be occluded within a stem loop in all three structures.
